# Engineering Dual-Input Glucose- and Temperature-Sensitive Lysis Circuits in *Corynebacterium glutamicum* for Efficient Intracellular Product Recovery

**DOI:** 10.3390/microorganisms13122758

**Published:** 2025-12-04

**Authors:** Ziyu Ye, Shihui Wang, Qiyue Wang, Liming Ouyang, Youyuan Li, Lixin Zhang

**Affiliations:** State Key Laboratory of Bioreactor Engineering, School of Biotechnology, East China University of Science and Technology, Shanghai 200237, China; yzyemail0518@163.com (Z.Y.); 19834406047@163.com (S.W.); 18700973359@163.com (Q.W.); ouyanglm@ecust.edu.cn (L.O.); lxzhang@ecust.edu.cn (L.Z.)

**Keywords:** *Corynebacterium glutamicum*, programmable cell lysis, holin–endolysin system, glucose-responsive promoter, temperature-inducible system, dual-input *AND-gate* circuit

## Abstract

*Corynebacterium glutamicum* is a versatile microbial cell factory, but efficient recovery of intracellular macromolecules remains a major challenge. In this study, we engineered environmentally controllable lysis systems to enable programmable product release. A glucose-responsive module, combining the *cg3195* promoter with phage-derived holin–endolysin genes (*clg51-50*), triggered lysis when extracellular glucose dropped below 0.19–0.36 g/L. A separate temperature-inducible system employing the *cI857-CJ1OX2* module activated lysis at 42 °C. These modules were further integrated into a dual-input *AND-gate* circuit, enhancing regulatory precision and suppressing premature lysis, with additional operator copies allowing temporal tuning of induction. Functional validation using fluorescence, cell density measurements, and scanning electron microscopy confirmed robust, tunable responses under defined environmental cues. Collectively, these programmable lysis systems demonstrate that stimulus-responsive genetic circuits can be rationally designed to control cell disruption, providing a promising approach to streamline downstream processing and reduce extraction costs in industrial fermentation of *Corynebacterium glutamicum*.

## 1. Introduction

*Corynebacterium glutamicum* (*C. glutamicum*) is an important industrial microbial cell factory that is now widely used to produce a diverse range of value-added chemicals and biofuels, going beyond its traditional role in amino acid production [1]. Given the pressing environmental challenges, including limited fuel resources and climate change, metabolic engineering *C. glutamicum* to convert alternative renewable feedstocks into more valuable products has also become a major focus of current research [2].

Among these bio-products, some are easily released from *C. glutamicum*, such as amino acids [3], organic acids [4] and alcohols [5]. However, some high-molecular weight compounds, such as fatty acid [6] cannot be readily released from the cells. To extract these macromolecules, cell lysis is often a necessary step [7]. A variety of cell lysis methods are available, which can be roughly divided in mechanical and non-mechanical approaches [8]. While all conventional methods are currently used for cell lysis in various applications, they have certain disadvantages that limit their broader applicability [9], including high energy consumption, complex operation or product degradation. In particular, extraction costs remain a major issue, as traditional methods for recovering products from bacterial cells are reported to represent 20–30% of the total production cost. Compared with bacteria, the downstream processing costs of microalgae are even higher, representing 70–80% of overall expenses, primarily due to their thicker and more complex cell walls containing hemicellulose and other polysaccharides [10].

To reduce extraction costs and increase product yields, attempts have been made to release valuable bio-products by lysing host cells in a genetically regulated manner. Liu and Curtiss designed and constructed a nickel-inducible lysis system in *Synechocystis* sp. PCC 6803 to facilitate lipids extraction for biofuel production [11]. Their results demonstrated that applying a phage-derived holin–endolysin lysis strategy enables precise and immediate cell disruption in cyanobacteria. Similarly, by placing the lysis gene under the control of an improved light-responsive regulatory system in *Escherichia coli* (*E. coil*), Wang et al. successfully controlled cell lysis by switching light on or off [12]. These findings indicate that lysis systems can be engineered to respond to diverse environmental signals. To date, most of the developed lysis systems have been implemented in algae and *E. coli*, while similar systems in *C. glutamicum* remain largely unexplored.

In this study, we aimed to design and construct a controllable lysis system in *C. glutamicum* to facilitate extracting bio-products from cells. Using this system, the strain can respond to changes in glucose concentration and temperature prior to the completion of fermentation to achieve spontaneous lysis. Two independent lysis systems were successfully characterized, each activated specifically when glucose levels fell below 0.19–0.36 g/L or when the temperature exceeded 42 °C. These sensing modules were further integrated through an *AND-logic* gate to form a dual-control system with enhanced regulatory precision.

## 2. Materials and Methods

### 2.1. Bacterial Strains and Growth Conditions

All strains used in this study are listed in Table 1. *E. coli* TOP 10 was used for cloning and cultivated in LB medium. *C. glutamicum* ATCC 13032 (Accession: BX927147) was used as a wild-type strain. This wild type strain and all derived *C. glutamicum* were routinely grown in BHIS medium containing 37 g/L brain heart infusion (Becton, Dickinson and Company, Detroit, MI, USA) supplemented with 91 g/L sorbitol (Macklin, Shanghai, China). Nalidixic acid (50 mg/L for *C. glutamicum*) and kanamycin (50 mg/L for *E. coli*, 25 mg/L for *C. glutamicum*) were added when necessary.

To cultivate *C. glutamicum* recombinant strains, a loopful of cells from a single colony grown on a BHIS agar plate was used to inoculate 25 mL of CASO medium as a preculture, followed by incubation at 30 °C for 16–18 h. The cells were then harvested and washed twice with CASO medium before inoculation into fresh medium at an initial OD_600_ of 0.3. For glucose concentration–response experiments, cultures were supplemented with different initial glucose concentrations. For lytic protein expression, 0.1 mM IPTG was added during the exponential growth phase. In temperature-induced expression assays, cells were first cultivated at 30 °C for 4–5 h and then shifted to 42 °C for induction. When appropriate, antibiotics (nalidixic acid, 50 mg/L; kanamycin, 25 mg/L) were added as required.

### 2.2. Construction of Plasmids and Transformation

All plasmids used in this study are listed in Table 1. The corresponding plasmids were synthesized by GenScript (Nanjing, China). *C. glutamicum* was transformed by electroporation following the method described by Liebl et al. [13]. All recombinant strains obtained through transformation were subsequently verified by colony PCR and DNA sequencing to confirm the integrity of the target sequences (Sangon Biotech, Shanghai, China).

### 2.3. Analysis Methods

Culture samples were withdrawn from the flasks and diluted in CASO medium to an OD_600_ below 1.0 prior to fluorescence analysis, which was performed using a CLARIOstar multimode microplate reader (BMG Labtech, Ortenberg, Germany) equipped with a 570 ± 15 nm excitation filter and 620 ± 20 nm emission filter. Glucose concentrations were determined using a CheKine™ micro glucose assay kit (Abbkine, Wuhan, China).

For scanning electron microscopy (SEM), bacterial cells were fixed with 2.5% (*v*/*v*) glutaraldehyde (Macklin, Shanghai, China) and dehydrated following the procedure described by Hünnefeld et al. [14]. The prepared samples were analyzed using a scanning electron microscope (Hitachi High-Technologies Corporation, Tokyo, Japan) operated at an acceleration voltage of 15 or 30 kV under high vacuum.

### 2.4. Statistical Analysis

All data are presented as the means ± standard deviation Student’s *t*-test was used for pairwise comparisons, and *p* < 0.05 was considered statistically significant.

## 3. Results

### 3.1. Construction and Characterization of a Glucose-Responsive Genetic Element

There are currently few reports on glucose concentration–responsive biosensors in *C. glutamicum*. This study focused on a reporter gene-based single-cell cAMP biosensor [15], which consists of two main components: the promoter of the *cg3195* gene (*P*_cg3195_), and the reporter gene *eyfp*. This promoter is repressed by the GlxR–cAMP complex and responds to intracellular concentrations of both GlxR and cAMP.

As a global transcriptional regulator in *C. glutamicum*, GlxR was initially characterized as a repressor of the *aceB* promoter, which encodes malate synthase in the glyoxylate shunt [16]. Its DNA-binding motif exhibits a high degree of similarity to that of the cAMP receptor protein (CRP) in *E. coli* [17]. GlxR has been shown to modulate the expression of approximately 200 genes in *C. glutamicum* in a cAMP-dependent manner [18]. Notably, the expression of *glxR* itself is not affected by the type of carbon source [16]. For instance, during growth in glucose medium, *glxR* mRNA levels remain relatively stable between the logarithmic and stationary phases [19]. In contrast, intracellular cAMP concentrations are highly sensitive to carbon source availability [20]. When glucose serves as the sole carbon source, cAMP levels gradually decline as glucose is depleted [16]. Based on these findings, the GlxR–cAMP regulatory system represents a promising candidate for engineering a glucose-responsive genetic element.

According to Schulte et al. [15], a 188 bp fragment of the *P*_cg3195_ spanning 163 bp upstream and 24 bp downstream of the transcription start site was fused to the reporter gene *mCherry*. In this study, we selected *mCherry* instead of *eyfp* to minimize background fluorescence originating from the host cells and culture medium (Supplementary Figure S1). The native ribosome-binding site of *P*_cg3195_ was maintained at the same distance from the start codon of *mCherry* as it was from the start codon of *cg3195* in the genome. The resulting fragment was cloned into the *E. coli–C. glutamicum* shuttle expression vector pZ8-ptac, yielding the plasmid pZ8-Pcg3195-mcherry.

**Figure 1:**
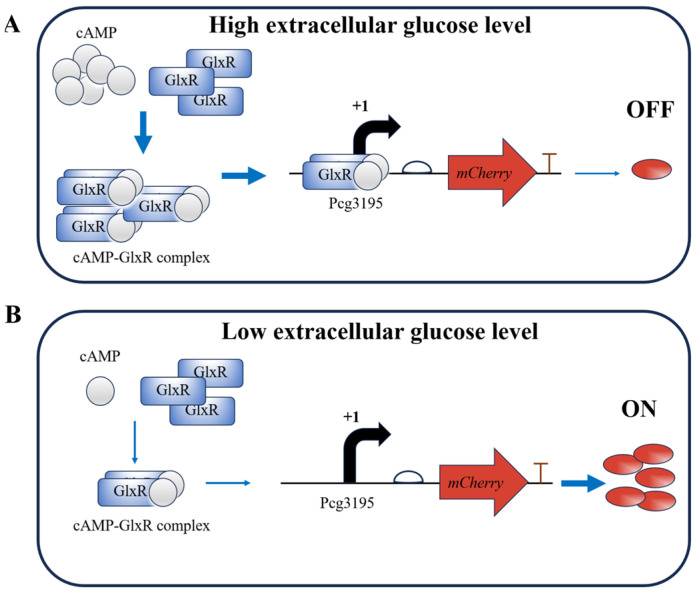
Schematic representation of the glucose-responsive element in *C. glutamicum*. Arrows are used as follows: bent black arrows for transcription direction from the start site; straight blue arrows for causal relationships or processes leading to subsequent events; and red arrows for the position and transcription direction of the *mCherry* gene. (**A**) High extracellular glucose concentrations promote the formation of the cAMP–GlxR complex, which binds to the *cg3195* promoter and represses expression of the *mCherry* reporter gene. (**B**) Low extracellular glucose concentrations result in reduced levels of the cAMP–GlxR complex and increased reporter gene expression.

The expected principle of the glucose-responsive element in *C. glutamicum* is illustrated in Figure 1. High initial extracellular glucose elevates intracellular cAMP levels, promoting the formation of GlxR–cAMP complexes that repress *mCherry* expression. As fermentation proceeds and extracellular glucose concentrations gradually decrease, this repression is relieved.

**Figure 2:**
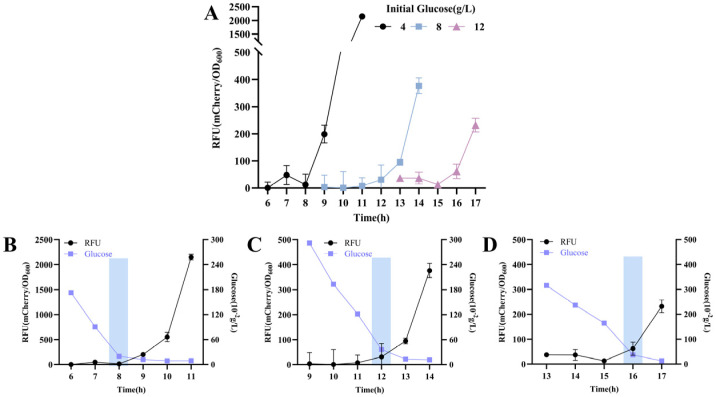
Dynamic response of the glucose-responsive element to glucose depletion in strain CGY1. (**A**) Time–relative fluorescence unit (RFU) profile in strain CGY1 cultured with different initial glucose concentrations. Time–RFU–glucose concentration profile at an initial glucose concentration of (**B**) 4 g/L, (**C**) 8 g/L, and (**D**) 16 g/L. Rectangular boxes in all panels indicate the occurrence of fluorescence inflection points.

To evaluate the response of the element to altered extracellular glucose levels, the wild-type *C. glutamicum* strain carrying plasmid pZ8-Pcg3195-mcherry (Strain CGY1) was cultivated in CASO medium supplemented with varying glucose concentrations between 4 and 16 g/L. As shown in Figure 2, *mCherry* expression increased significantly following identifiable inflection points—a trend that aligns with our preliminary data (Supplemental Figure S2). These inflection points occurred at later stages with increasing initial glucose concentrations (4 g/L: 8 h; 8 g/L: 12 h; 16 g/L: 16 h). Notably, the glucose concentrations stabilized near these points within a narrow range (0.19–0.36 g/L) across all groups, indicating that downstream reporter gene expression was gradually triggered upon reaching this threshold. It is also noteworthy that the recombinant strain maintained readily detectable *mCherry* expression even when glucose concentrations were above the threshold, suggesting the presence of promoter leakage in the response element, which may represent a potential limitation for practical applications.

### 3.2. Construction and Functional Evaluation of Phage-Derived Lysis Elements

To achieve cell lysis, two groups of phage-derived genes were selected as lysis elements. The first was *cg1974*, annotated as a potential lysozyme and derived from the prophage CGP3 of *C. glutamicum* ATCC 13032 [21]. The second group consisted of two overlapping genes, *clg51* and *clg50*, from phage CL31 of *C. glutamicum* ATCC 15059, which are predicted to encode holin and lytic proteins [14].

**Figure 3:**
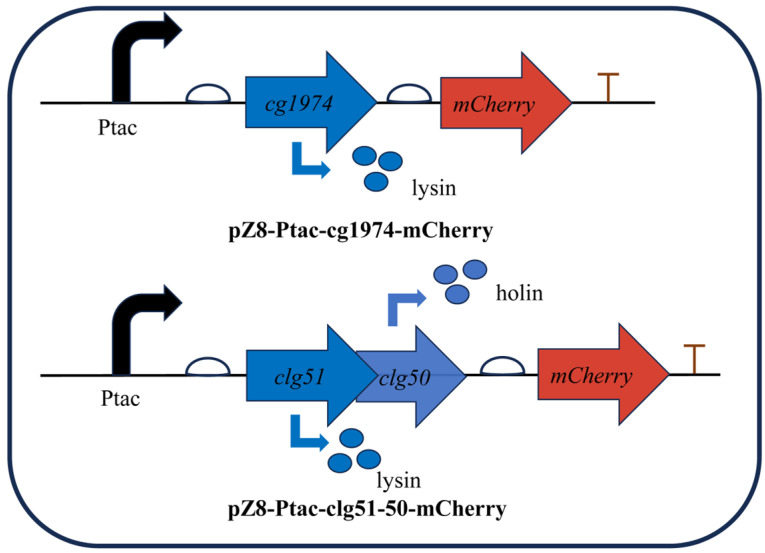
Schematic representation of inducible lysis cassettes used to validate the function of phage-derived lysis genes. Expression of the lytic proteins is induced by the addition of IPTG. Bent black arrows indicate the direction of transcription from the start site; bent blue arrows represent the translation process from gene to protein; straight arrows denote the position and transcription direction of three genes (*clg51*, *clg50* and *mCherry*).

To validate the lytic activity of these elements, the lysis genes were fused to the reporter gene *mCherry* and cloned downstream of the IPTG-inducible *tac* promoter on the plasmid pZ8-Ptac (Figure 3). The resulting plasmids were then transformed into wild-type *C. glutamicum* to generate strains CGY2::pZ8-Ptac-cg1974-mCherry and CGY3::pZ8-Ptac-clg51-50-mCherry, respectively.

**Figure 4:**
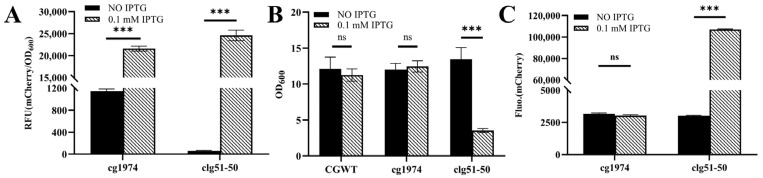
Functional characterization of phage-derived lysis elements in *C. glutamicum*. (**A**) RFU of recombinant strains expressing lytic proteins after 24 h of induction with or without 0.1 mM IPTG. (**B**) Changes in cell density following IPTG induction. (**C**) Fluorescence intensity in the culture supernatant. A significant increase in extracellular fluorescence is observed only in the IPTG-induced CGY3 culture. Statistical significance was determined using Student’s *t*-test: *** *p* < 0.001; ns (not significant), *p* ≥ 0.05.

The strains were cultivated in CASO medium, and 0.1 mM IPTG was added during the logarithmic growth phase to induce lysis gene expression. As shown in Figure 4A, the fluorescence of both strains increased substantially in the presence of IPTG compared to the uninduced control after 24 h of incubation.

However, following IPTG induction, the two strains exhibited distinct phenotypic characteristics (Figure 4B). The culture broth of CGY2 remained similarly turbid to that of the wild-type control, with no significant change in biomass. In contrast, the culture of CGY3 became viscous and clear after induction, accompanied by a marked reduction in biomass (a 74% decrease in OD_600_). These observations suggest that the lytic proteins encoded by *clg51-50* may mediate host cell lysis.

To further confirm this hypothesis, the supernatants of the cultures were analyzed. As shown in Figure 4C, only the induced CGY3 group exhibited a substantial increase in fluorescence intensity in the supernatant—34.5 times higher than that of the uninduced control. This pattern is consistent with the characteristics of a holin–endolysin lysis system, in which disruption of cell wall integrity leads to the release of intracellular contents, such as fluorescent proteins, into the culture medium. In comparison, the single endolysin encoded by *cg1974* did not cause significant lysis, suggesting that it may require additional co-factors to exert its lytic function.

**Figure 5:**
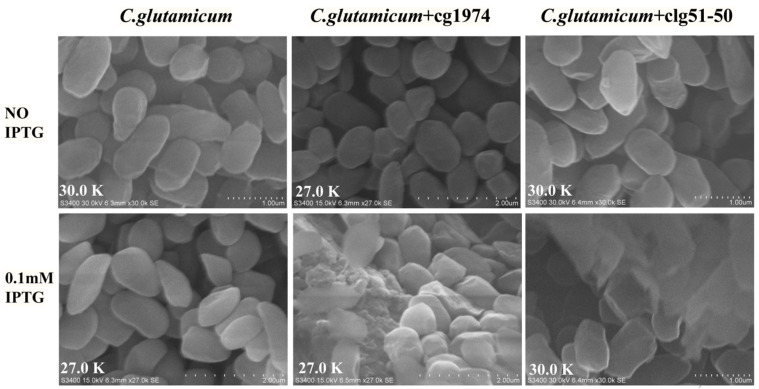
SEM images of *C. glutamicum* cells following induction with 0.1 mM IPTG, captured at 27,000× and 30,000× magnification.

SEM images (Figure 5) were further used to visualize IPTG-induced cell lysis. As shown in the figures, normal cells were structurally intact, appearing as short rods or bars. In contrast, CGY3 harboring plasmid pZ8-Ptac-clg51-50-mCherry exhibited a pronounced central cleft after IPTG induction, indicating successful cell lysis. CGY2 harboring plasmid pZ8-Ptac-cg1974-mCherry, however, displayed only minor surface depressions with no obvious cleavage. Based on these observations, the overlapping genes *clg51-50* were selected as the lysis element for subsequent experiments.

### 3.3. Construction and Validation of a Controllable Glucose-Responsive Lysis System

The characterized glucose-responsive element (*P*_cg3195_) and the lysis element (overlapping genes *clg51-50*) were assembled to construct a controllable lysis system capable of responding to extracellular glucose levels in *C. glutamicum*. Using plasmid pZ8-Pcg3195-*mCherry* as the backbone, the lysis element was precisely inserted between *P*_cg3195_ and the *mCherry* reporter gene. A ribosome-binding site (5′-AAAGGAGGACAACC-3′) was included at the 3′ end of the lysis element to maintain translation efficiency of *mCherry*, resulting in the recombinant plasmid pZ8-Pcg3195-clg51-50-mCherry.

**Figure 6:**
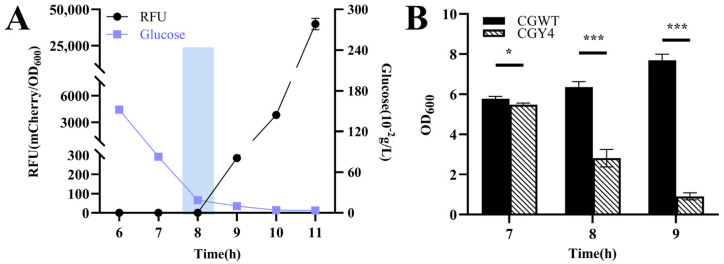
Validation of the controllable glucose-responsive lysis system in *C. glutamicum* CGY4. (**A**) Time–RFU–glucose concentration profile at an initial glucose concentration of 4 g/L. The rectangular box indicates the fluorescence inflection point at 8 h, corresponding to glucose depletion to the critical threshold (~0.19 g/L). (**B**) Magnified view of cell density changes for CGY4 and the wild-type control during the critical transition period. Statistical significance was determined using Student’s *t*-test: * *p* < 0.05, *** *p* < 0.001.

The strain CGY4 harboring pZ8-Pcg3195-clg51-50-mCherry was inoculated into CASO medium with an initial glucose concentration of 4.00 g/L. A distinct inflection point at 8 h was observed, corresponding to a significant increase in *mCherry* expression (Figure 6A). At this time, the extracellular glucose concentration was approximately 0.19 g/L, which falls within the previously characterized threshold. Analysis of cell density during this critical window (Figure 6B) revealed a sharp decline compared to the continuously increasing density of the wild-type control. These results confirm that the constructed lysis system can trigger spontaneous cell lysis in response to changes in extracellular glucose concentration.

### 3.4. Construction and Validation of a Temperature-Responsive Lysis System

Using a similar strategy, a temperature-responsive lysis system was engineered in *C. glutamicum* by integrating the characterized lysis element with the heat-inducible system developed by Jo et al. [22]. The core of this system is the hybrid promoter *CJ1OX2* (128 bp), which comprises a 52 bp native *CJ1* promoter from *Corynebacterium ammoniagenes* and two tandem repeats of the 17 bp *OL1* operator sequence derived from the *PL* promoter of λ bacteriophage.

To construct the temperature-inducible expression vector, this hybrid promoter fragment replaced the original *tac* promoter and *lacI* gene in pZ8-Ptac-clg51-50-mCherry, thereby placing the expression of the lytic protein and *mCherry* under the control of *CJ1OX2*. Subsequently, a 333 bp expression cassette encoding the temperature-sensitive repressor *cI857* was inserted in reverse orientation upstream of the hybrid promoter, resulting in pZ8-cI857-CJ1OX2. As the *lacI* gene had been replaced, *cI857* was constitutively expressed under the control of the *tac* promoter.

**Figure 7:**
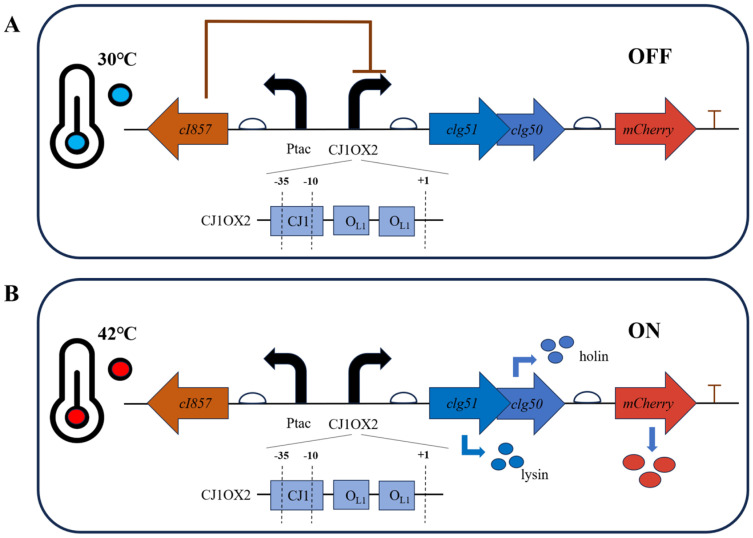
Design principle of the temperature-responsive lysis system in *C. glutamicum*. Bent black arrows indicate the direction of transcription from the start site; bent blue arrows represent the translation process from gene to protein; straight arrows denote the position and transcription direction of four genes (*cI857*, *clg51*, *clg50* and *mCherry*). (**A**) At the permissive temperature (30 °C), the functional *cI857* repressor binds to the *OL1* operators within the hybrid promoter *CJ1OX2*, preventing transcription of the downstream lysis genes (*clg51-50*) and the *mCherry* reporter gene. (**B**) Upon heat induction at 42 °C, the temperature-sensitive cI857 repressor is inactivated and dissociates from the promoter, allowing RNA polymerase to access CJ1OX2 and induce gene expression.

The anticipated behavior of this temperature-responsive lysis system is illustrated in Figure 7. At 30 °C, the temperature-sensitive cI857 repressor binds to the *OL1* operators, effectively repressing downstream gene expression (Figure 7A). Upon shifting the temperature to 42 °C, the repressor is inactivated, leading to induction of lytic protein and *mCherry* expression (Figure 7B).

**Figure 8:**
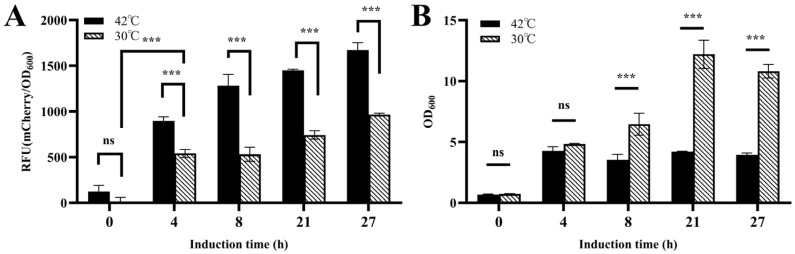
Functional validation of the temperature-responsive lysis system in *C. glutamicum* CGY5. (**A**) Time–relative fluorescence unit (RFU) profile, and (**B**) cell density changes of CGY5 with or without heat induction. Statistical significance was determined using Student’s *t*-test: *** *p* < 0.001; ns (not significant), *p* ≥ 0.05.

To validate the temperature-inducible lysis system in the engineered strain CGY5 harboring pZ8-cI857-CJ1OX2, its behavior was compared under continuous cultivation at 30 °C (control) and after a temperature shift to 42 °C (Figure 8A). The results demonstrated that after 4 h of induction at 42 °C, fluorescence in the induced group was significantly higher than in the control, indicating that the cI857 repressor effectively de-repressed the *CJ1OX2* promoter upon heating, leading to activated *mCherry* expression. A basal level of expression leakage was also observed at 30 °C. Furthermore, after 8 h of induction, the cell density of the induced group was significantly lower than that of the control (Figure 8B), suggesting concomitant activation of the lysis module under the same conditions. These results confirm that the temperature-responsive lysis system responds appropriately to heat induction to achieve cell lysis.

### 3.5. Dual-Cascade Regulatory Lysis System: Construction, Validation, and Temporal Dynamics

To further optimize the performance of the two environment-responsive lysis systems, a dual-cascade regulatory strategy was developed based on the Boolean *AND-gate* principle. For this purpose, the glucose-sensing promoter *P_cg3195_* and the heat-sensing regulatory module *cI857–OL1* were functionally connected in series. This design employed a *roadblock* repression mechanism derived from *Escherichia coli* [23], which was implemented by introducing multiple binding sites (*OL1*) for the constitutively expressed cI857 repressor downstream of the promoter. The bound repressors then serve as a physical obstacle to RNA polymerase, thereby achieving enhanced transcriptional repression and enabling the establishment of a novel and tightly regulated lysis system.

**Figure 9:**
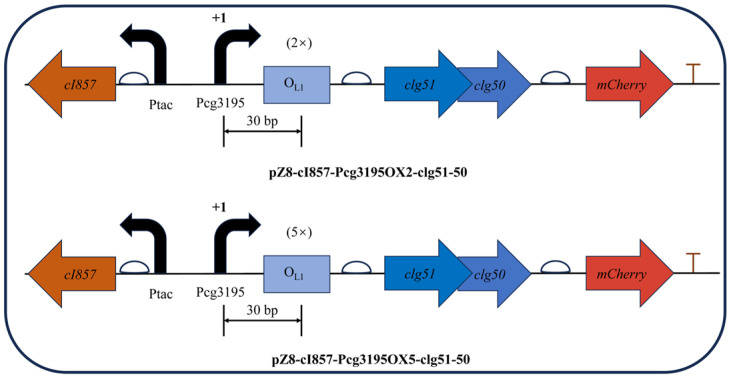
Schematic representation of the dual-cascade regulatory lysis systems based on the Boolean *AND*-*gate* principle. The glucose-sensing promoter (*P*_cg3195_) and the heat-inducible regulatory module (*cI857*–*OL1*) were arranged in series to achieve two-level control of the phage-derived lysis genes (*clg51-50*). The design enables expression only under simultaneous glucose depletion and elevated temperature.

Building upon the validated temperature-responsive vector pZ8-cI857-CJ1OX2, a modified construct was generated by replacing the *CJ1* promoter with the *cg3195* promoter, while retaining the upstream *cI857* repressor expression cassette. Considering the DNA footprint of an elongating RNA polymerase, two (38 bp in total) or five (101 bp in total) *OL1* operator sequences were precisely inserted 30 bp downstream of the *P_cg3195_* transcription start site (TSS, +1) within the non-coding region. The resulting recombinant plasmids, pZ8-cI857-Pcg3195OX2-clg51-50 and pZ8-cI857-Pcg3195OX5-clg51-50, maintained the structural integrity of the downstream *clg51*-*50*–*mCherry* expression cassette (Figure 9).

The dual-signal response system was designed to activate lysis gene expression only when two environmental conditions were simultaneously satisfied: (i) the extracellular glucose concentration dropped below the defined threshold, and (ii) the temperature increased to the induction level. To validate this regulatory logic, fermentation experiments were performed in CASO medium with an initial glucose concentration of 8.00 g/L, and the critical time window between 10 h and 16 h was selected for analysis based on prior empirical observations.

**Figure 10:**
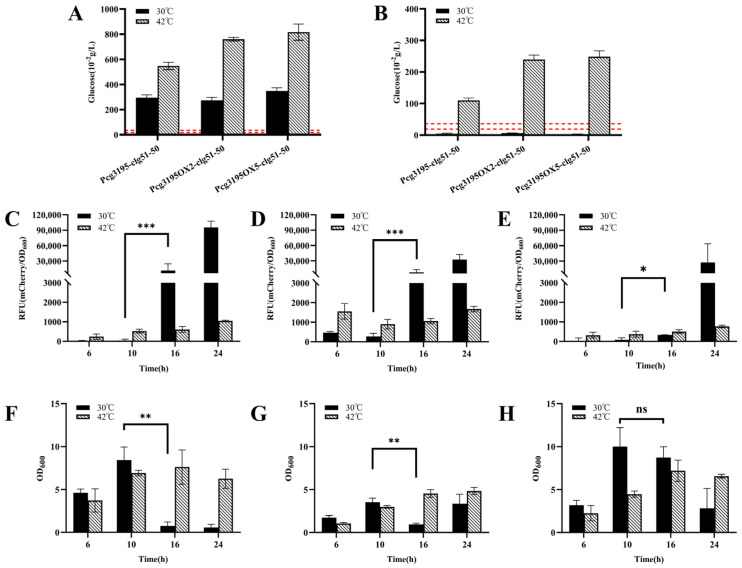
Functional validation of the dual-cascade regulatory lysis systems in *Corynebacterium glutamicum*. (**A**,**B**) Extracellular glucose concentration profiles of the recombinant *C. glutamicum* strains at 10 h (**A**) and 16 h (**B**) under constant 30 °C or after a temperature shift to 42 °C at 6 h. The dashed line indicates the characterized glucose threshold (0.19–0.36 g/L). (**C**–**E**) Time–RFU profiles for the three recombinant strains—CGY*4* (**C**), CGY6 (**D**), and CGY7 (**E**)—cultivated with or without heat induction. Only the strain harboring five *OL1* operators (CGY7, panel (**E**)) exhibited effective suppression of premature expression leakage following glucose depletion. (**F**–**H**) Corresponding cell density (OD_600_) profiles for the same strains under both thermal conditions. The absence of a significant OD_600_ decline in panel H correlates with the suppressed fluorescence observed in panel E, confirming the enhanced transcriptional repression achieved by the OX5 construct. Statistical significance was determined using Student’s *t*-test: * *p* < 0.05, ** *p* < 0.01, *** *p* < 0.001; ns (not significant), *p* ≥ 0.05.

As shown in Figure 10A, during cultivation at 30 °C, the glucose concentration in the broth of all three recombinant strains decreased below the threshold by 16 h. However, both *mCherry* fluorescence measurements (Figure 10C–E) and changes in cell density (Figure 10F,G) indicated that the lysis module was activated even in the absence of temperature induction, which deviated from the intended *AND-gate* control design. In contrast, when a temperature upshift to 42 °C was applied at 6 h, the residual glucose concentration at 16 h remained significantly higher than the threshold (Figure 10B), and the bacterial growth continued to increase (Figure 10F–H), implying that the glucose-responsive switch was not triggered at this stage.

It was further verified (Supplemental Figure S3) that despite a modest reduction in the transcriptional activity of *Pcg3195* at 42 °C, the promoter retained its correct response to glucose concentration, and the function of the lysis proteins was largely unaffected. Collectively, these results suggest that premature temperature induction at 6 h disrupted normal cell growth and metabolism, leading to incomplete glucose consumption and consequently masking the expected activation of the dual-responsive lysis system.

Further analysis under non-induced conditions (30 °C) (Figure 10E,H) revealed that compared with the other two strains (CGY6::pZ8-cI857-Pcg3195OX2-clg51-50 and CGY4::pZ8-Pcg3195-clg51-50-mCherry), the strain CGY7, which carried a higher copy number of the operator *OL1* (OX5), showed no significant increase in *mCherry* fluorescence and no notable reduction in biomass at 16 h, despite the glucose concentration having dropped below the threshold (Figure 10B). These results suggest that increasing the copy number of the *OL1* repressor operator downstream of the promoter may effectively delay the expression of the lytic protein.

## 4. Discussion

In this study, two independent environmentally responsive lysis systems were successfully constructed and validated in *C. glutamicum*. Each system was designed to trigger autonomous cell lysis specifically in response to either glucose depletion (below 0.19–0.36 g/L) or a temperature upshift to 42 °C. Building upon these modules, a dual-input, *AND-gate* cascade regulatory circuit was developed to integrate both environmental cues. This design effectively minimized leaky expression under non-inducing conditions by requiring the simultaneous occurrence of low glucose and elevated temperature for activation. The introduction of additional *OL1* operator sequences into the dual-signal control system further demonstrated the potential to delay lysis induction. Notably, the strain harboring five operator copies (OX5) exhibited a pronounced delay in activation under non-inducing conditions, indicating that transcriptional repression can effectively modulate the temporal dynamics of gene expression.

A comparative analysis revealed that the holin–endolysin system (*clg51-50*) derived from phage CL31 exhibited markedly higher lytic efficiency than the single endolysin *cg1974* from phage CGP3. In most double-stranded DNA phages, host cell lysis is accomplished through the coordinated action of a holin and an endolysin. The holin forms pores in the cytoplasmic membrane, facilitating the translocation of endolysin into the periplasmic space, where it hydrolyzes the peptidoglycan layer to enable rapid and precisely timed cell lysis [24]. Although some endolysins can be exported via holin-independent pathways [25]—for example, through signal–anchor–release (SAR) peptides [26] or by interacting with host-derived components such as teichoic acids [27]—the low lytic activity of *cg1974* when expressed alone suggests the absence of an efficient transport mechanism. Notably, no canonical holin gene has been annotated within the CGP3 genome; however, several open reading frames predicted to encode proteases or hydrolases may assist in the translocation or activation of *cg1974* [21]. Therefore, when designing heterologous lytic systems, the functional compatibility between endolysins and their cognate transport or activation partners must be carefully considered to ensure efficient bacterial cell disruption.

Further analysis of the temperature-inducible lysis system revealed a distinct temporal delay between the marked increase in *mCherry* expression and the subsequent decline in biomass. This observation suggests that the functional execution of lytic proteins occurs later than the maturation of the fluorescent reporter. The observed time lag can be attributed to two principal factors. First, holin accumulation on the cytoplasmic membrane must reach a critical threshold before oligomerization and pore formation can occur—a process governed by concentration-dependent kinetics. Second, endolysin must undergo proper post-translational folding, translocation to the cell wall, and catalytic hydrolysis of peptidoglycan, all of which are sequential biological events requiring additional time. This regulatory pattern is consistent with the well-established *“molecular clock”* mechanism of phage-mediated lysis, in which holin accumulation and oligomerization temporally coordinate the release and activation of endolysin [28,29]. Therefore, when applying such lysis systems in industrial fermentation processes, it is crucial to consider this inherent time lag. By precisely determining the induction point, the dynamic balance between product synthesis and cell lysis can be optimized, thereby maximizing both yield and recovery efficiency of the desired bio-product.

The development of the dual-signal cascade regulatory system provides a promising framework for achieving precise and autonomous control of cell lysis in *C. glutamicum*. By integrating glucose depletion and temperature elevation as dual inputs, the system effectively minimizes leaky expression under non-inducing conditions while ensuring robust activation only when both environmental cues are satisfied. This Boolean *AND-gate* architecture not only enhances the controllability of the lytic response but also offers modular flexibility for integrating additional regulatory elements, such as metabolite sensors or quorum-sensing modules, to further refine temporal and spatial precision. Moreover, the incorporation of multiple operator sites downstream of the promoter significantly improved transcriptional repression strength, thereby delaying premature lytic activation. The strain carrying five *OL1* copies (OX5) exhibited a clear postponement in lysis onset under non-induced conditions, demonstrating that fine-tuning operator copy number can effectively adjust the threshold and timing of system activation. Nonetheless, residual background expression indicates that complete repression remains challenging. Future optimization could focus on balancing repressor abundance and operator affinity, or on employing advanced regulatory strategies—such as CRISPR interference or orthogonal repressors—to achieve tighter control.

From an industrial perspective, such programmable autolysis systems have the potential to substantially streamline downstream recovery of intracellular products by enabling synchronized cell disruption without external chemical or mechanical treatment. This self-regulated strategy not only simplifies bioprocessing but also enhances product purity and reduces environmental impact, providing a sustainable and scalable solution for microbial manufacturing. Coupling programmable lysis circuits with production pathways could allow for simultaneous synthesis and recovery of high-value intracellular products, maximizing yield while minimizing downstream processing costs. Collectively, these findings underscore the potential of synthetic regulatory systems to advance sustainable and efficient microbial biomanufacturing, offering a versatile platform for future industrial and biotechnological applications.

## Figures and Tables

**Table 1:** Bacterial strains and plasmids used in this work.

Strain or Plasmid	Description	Source
Strain		
*E. coli* TOP 10	F-mcrAΔ(mrr-hsdRMS-mcrBC) φ80lacZΔM15ΔlacX74recA1 araΔ139Δ(ara-leu)7697 galU galK rpsL (Str^R^)endA1nupG	Laboratory
*C. glutamicum* ATCC 13032	Wild-type	ATCC
CGY1	*C. glutamicum* ATCC 13032 harboring the pZ8-Pcg3195-mCherry	This work
CGY2	*C. glutamicum* ATCC 13032 harboring the pZ8-Ptac-cg1974-mCherry	This work
CGY3	*C. glutamicum* ATCC 13032 harboring the pZ8-Ptac-clg51-50-mCherry	This work
CGY4	*C. glutamicum* ATCC 13032 harboring the pZ8-Pcg3195-clg51-50-mCherry	This work
CGY5	*C. glutamicum* ATCC 13032 harboring the pZ8-cI857-CJ1OX2	This work
CGY6	*C. glutamicum* ATCC 13032 harboring the pZ8-cI857- Pcg3195OX2-clg51-50	This work
CGY7	*C. glutamicum* ATCC 13032 harboring the pZ8-cI857- Pcg3195OX5-clg51-50	This work
Plasmid		
pZ8-Ptac	Km^R^, *E. coli–C.glutamicum* shuttle expression vector carrying the *tac* promotor	Laboratory
pZ8-Pcg3195-mCherry	Derived from pZ8-Ptac by replacing the *tac* promotor with the *P*_cg3195_, carrying the gene *mCherry*	This work
pZ8-Ptac-cg1974-mCherry	pZ8-Ptac carrying the genes *cg1974*, *mCherry*	This work
pZ8-Ptac-clg51-50-mCherry	pZ8-Ptac carrying the genes *clg51*, *clg50*, *mCherry*	This work
pZ8-Pcg3195-clg51-50-mCherry	pZ8-Pcg3195-mCherry carrying the genes *clg51*, *clg50*	This work
pZ8-cI857-CJ1OX2	Derived from pZ8-Ptac-clg51-50-mCherry by inserting the *cI857* expression cassette and swapping the *P_cg3195_* for the *CJ1OX2* to control the genes *clg51*, *clg50*, *mCherry*	This work
pZ8-cI857-Pcg3195OX2-clg51-50	Derived from pZ8-cI857-CJ1OX2 by replacing the *CJ1* promotor with the *P*_cg3195_ and inserting two *OL1* operator sites downstream of the *P*_cg3195_	This work
pZ8-cI857-Pcg3195OX5-clg51-50	Derived from pZ8-cI857-CJ1OX2 by replacing the *CJ1* promotor with the *P*_cg3195_ and inserting five *OL1* operator sites downstream of the *P*_cg3195_	This work

## Data Availability

The original contributions presented in this study are included in the article/Supplementary Materials. Further inquiries can be directed to the corresponding author.

## Supplementary Materials

The following supporting information can be downloaded at: https://www.mdpi.com/article/10.3390/microorganisms13122758/s1, Supplementary Figure S1: Background fluorescence of CGWT cells and CASO medium. The intrinsic fluorescence of *Corynebacterium glutamicum* ATCC 13032 (CGWT) cell suspensions at increasing OD_600_ values and of fresh CASO medium (OD_600_ = 0) was measured. Fluorescence intensity was recorded at five distinct wavelength pairs corresponding to GFP (470/510 nm), YFP (497/540 nm), RFP (548/588 nm), mCherry (570/620 nm), and CFP (430/480 nm) using a microplate reader with a fixed gain of 1500. Each data point represents the mean of three technical replicates (n = 3); Supplementary Figure S2: Dynamic response of the glucose-responsive element in strain CGY1 to depletion of varying initial glucose concentrations. The fluorescence inflection points, indicating activation of the *cg3195* promoter, are marked by arrows. The observation that higher initial glucose concentrations result in later inflection points demonstrates that the response of this genetic element is regulated by extracellular glucose levels; Supplementary Figure S3: Effect of cultivation temperature on the function of the glucose response element and lysis element in strain CGY4 (A) Time–RFU–glucose concentration profile under constant 30 °C or after a temperature shift to 42 °C at 7 h. The strain was cultivated at an initial glucose concentration of 8 g/L. The rectangular box indicates the fluorescence inflection point at 12 h, corresponding to glucose depletion to the critical threshold. (**B**) Corresponding cell density (OD_600_) profiles for the same strains under both thermal conditions. The sharp decline in cell density at 42 °C occurred 12 hours post the fluorescent inflection point, indicating that elevated temperature does not impair the lytic function of the protein. Statistical significance was determined using Student’s *t*-test: *** *p* < 0.001. The strain was cultivated at an initial glucose concentration of 8 g/L. The rectangular box indicates the fluorescence inflection point at 12 h, corresponding to glucose depletion to the critical threshold. (B) Corresponding cell density (OD_600_) profiles for the same strains under both thermal conditions. The sharp decline in cell density at 42 °C occurred 12 hours post the fluorescent inflection point, indicating that elevated temperature does not impair the lytic function of the protein.

## Abbreviations

The following abbreviations are used in this manuscript:

*C. glutamicum*	*Corynebacterium glutamicum*
*E. coil*	*Escherichia coli*
SEM	Scanning electron microscopy
RFU	Relative fluorescence units
