# Supplementary material for: Engineering Dual-Input Glucose- and Temperature-Sensitive Lysis Circuits in Corynebacterium glutamicum for Efficient Intracellular Product Recovery
Source: Microorganisms. 2025 Dec 4;13(12):2758. doi: 10.3390/microorganisms13122758 (PMC12735821; doi:10.3390/microorganisms13122758)
Supplement: Supplementary file 1 [file microorganisms-13-02758-s001.zip › microorganisms-3956182-supplementary.pdf]

Supplementary Material

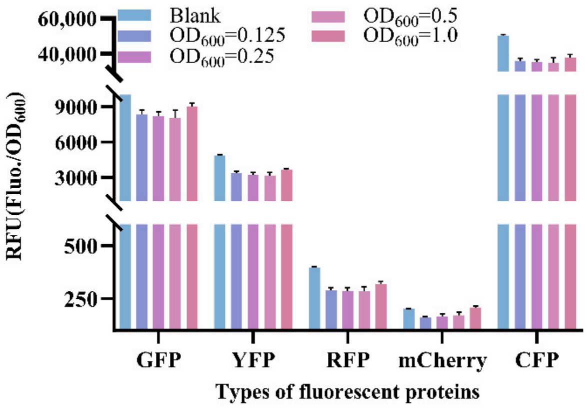

Supplementary Figure S1. Background fluorescence of CGWT cells and CASO medium. The intrinsic fluorescence of *Corynebacterium glutamicum* ATCC 13032 (CGWT) cell suspensions at increasing OD_{600} values and of fresh CASO medium (OD_{600} = 0) was measured. Fluorescence intensity was recorded at five distinct wavelength pairs corresponding to GFP (470/510 nm), YFP (497/540 nm), RFP (548/588 nm), mCherry (570/620 nm), and CFP (430/480 nm) using a microplate reader with a fixed gain of 1500. Each data point represents the mean of three technical replicates (n = 3).

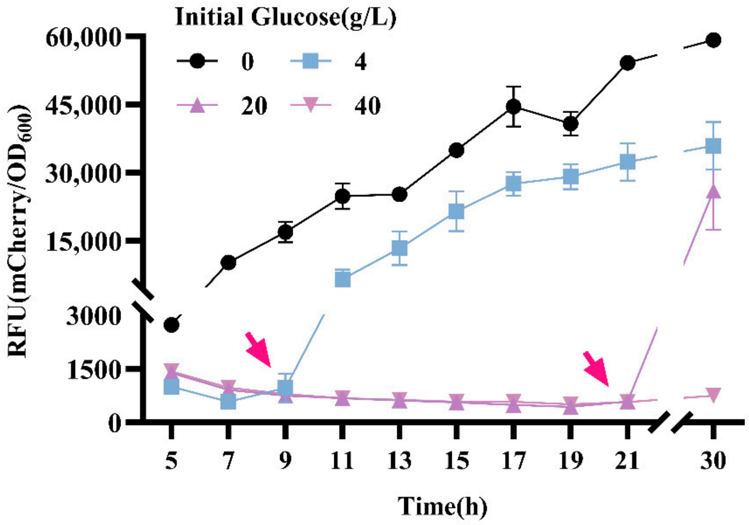

Supplementary Figure S2. Dynamic response of the glucose-responsive element in strain CGY1 to depletion of varying initial glucose concentrations. The fluorescence inflection points, indicating activation of the *cg3195* promoter, are marked by arrows. The observation that higher initial glucose concentrations result in later inflection points demonstrates that the response of this genetic element is regulated by extracellular glucose levels.

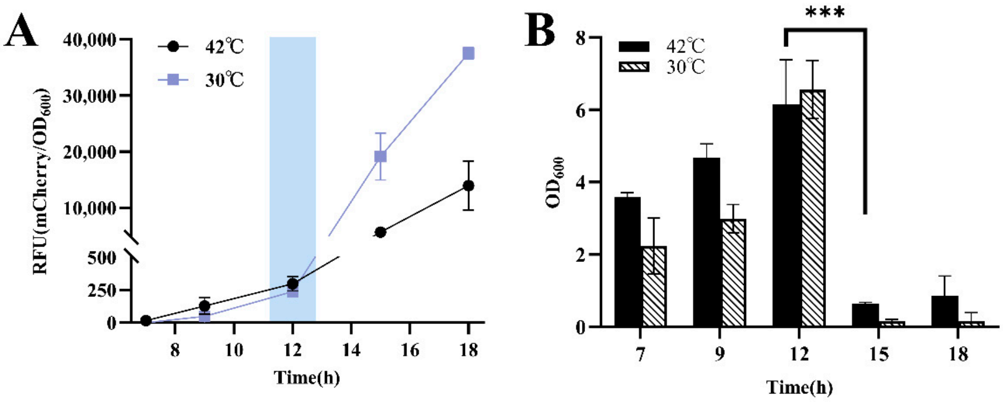

Supplementary Figure S3. Effect of cultivation temperature on the function of the glucose response element and lysis element in strain CGY4 (A) Time–RFU–glucose concentration profile under constant 30 °C or after a temperature shift to 42 °C at 7 h. The strain was cultivated at an initial glucose concentration of 8 g/L. The rectangular box indicates the fluorescence inflection point at 12 h, corresponding to glucose depletion to the critical threshold. (**B**) Corresponding cell density (OD_{600}) profiles for the same strains under both thermal conditions. The sharp decline in cell density at 42°C occurred 12 hours post the fluorescent inflection point, indicating that elevated temperature does not impair the lytic function of the protein. Statistical significance was determined using Student's *t*-test: *** $p < 0.001$.
